# Characterizations on a GRAS Electrospun Lipid–Polymer Composite Loaded with Tetrahydrocurcumin

**DOI:** 10.3390/foods13111672

**Published:** 2024-05-27

**Authors:** Zhenyu Lin, Jun Li, Qingrong Huang

**Affiliations:** 1 Department of Food Science, Rutgers University, 65 Dudley Road, New Brunswick, NJ 08901, USA; 2 College of Food Science, South China Agricultural University, Guangzhou 510642, China

**Keywords:** delivery system, tetrahydrocurcumin, electrospun, self-assemble, nano-emulsion

## Abstract

Electrospun/sprayed fiber films and nanoparticles were broadly studied as encapsulation techniques for bioactive compounds. Nevertheless, many of them involved using non-volatile toxic solvents or non-biodegradable polymers that were not suitable for oral consumption, thus rather limiting their application. In this research, a novel electrospun lipid–polymer composite (ELPC) was fabricated with whole generally recognized as safe (GRAS) materials including gelatin, medium chain triglyceride (MCT) and lecithin. A water-insoluble bioactive compound, tetrahydrocurcumin (TC), was encapsulated in the ELPC to enhance its delivery. Confocal laser scanning microscopy (CLSM) was utilized to examine the morphology of this ELPC and found that it was in a status between electrospun fibers and electrosprayed particles. It was able to form self-assembled emulsions (droplets visualized by CLSM) to deliver active compounds. In addition, this gelatin-based ELPC self-assembled emulsion was able to form a special emulsion gel. CLSM observation of this gel displayed that the lipophilic contents of the ELPC were encapsulated within the cluster of the hydrophilic gelatin gel network. The FTIR spectrum of the TC-loaded ELPC did not show the fingerprint pattern of crystalline TC, while it displayed the aliphatic hydrocarbon stretches from MCT and lecithin. The dissolution experiment demonstrated a relatively linear release profile of TC from the ELPC. The lipid digestion assay displayed a rapid digestion of triglycerides in the first 3–6 min, with a high extent of lipolysis. A Caco-2 intestinal monolayer transport study was performed. The ELPC delivered more TC in the upward direction than downwards. MTT study results did not report cytotoxicity for both pure TC and the ELPC-encapsulated TC under 15 μg/mL. Caco-2 cellular uptake was visualized by CLSM and semi-quantified to estimate the accumulation rate of TC in the cells over time.

## 1. Introduction

**Figure 1:**
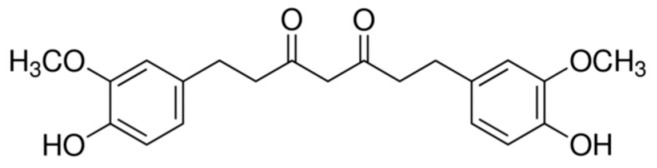
Chemical structure of TC.

TC (1,7-bis (4-hydroxy-3-methoxyphenyl) heptane-3,5-dione) is one of the major metabolites of curcumin detected in the intestine and liver area in humans and rats, and is related to the metabolism with NADPH-dependent curcumin reductase [1]. TC gained attention due to its potent anti-oxidative capability that is even superior to curcumin [2]. The chemical structure of TC is illustrated in Figure 1. Orally administered TC conjugates with glucuronides or sulfates catalyzed by glucuronyltransferases and sulfotransferases secreted via the liver to become tetrahydrocurcumin glucuronide corresponding monosulfate [3,4]. TC is the most potent antioxidant in the family of curcumin metabolites, substantiated by a series of anti-oxidative assays including linoleic acid autoxidation, rabbit erythrocyte ghost membrane peroxidation and rat liver microsome peroxidation [5]. Potential health benefits of orally administered TC include but are not limited to protection from hepatotoxicity [6], inhibition of cervical tumors [7], recovering the functions of carbohydrate metabolism-related enzymes and restoring the normal blood glucose level for diabetic rats [8], osteoarthritis relieving and reducing pro-inflammatory cytokines [9], anti-inflammation and anti-cancer [10], etc. Compared with curcumin, which is highly unstable under physiological and higher pH environments (90% lost at pH 7.2 in half an hour) [11], TC is much more compatible with the physiological environment (stable for 48 h at pH 7.0) and an even more harsh environment (about 68% retention after 48 h) [12]. This property should benefit the bioaccessibility and bioavailability of TC. However, the water solubility of TC is still very low, and thus the major part of orally consumed TC is unable to contact with digestive enzymes, thus achieving very low bioaccessibility.

To enhance the bioaccessibility of TC, researchers developed various types of delivery systems to encapsulate TC to be more compatible with the digestive environment. Solid lipid nanoparticles comprised of glyceryl monostearate as the major lipid, tween 80 as surfactant and soy lecithin as co-surfactant were reported for TC encapsulation, with a product particle size as small as 150 nm and over 80% loading efficiency. This study achieved in vitro release of TC up to 71.04% after 24 h [13]. Colloidal systems were also researched as an effective TC carrier. A calcium carbonate—β-cyclodextrin hybrid colloid system was utilized to load TC at 5.7 wt%. It was found to relieve dermal inflammatory situations in atopic dermatitis model mice while downregulating epithelial-related proinflammatory cytokines [14]. A lipid–polysaccharides system was another approach to entrap TC, which was carried out by dissolving TC in rapeseed oil to form an emulsion with a starch octenyl succinate/chitosan solution and then spray-dried or freeze-dried. It was observed to have anti-fungal effects against *Fusarium graminearum* [15]. Organo-emulsion gel generated from an emulsion of GRAS level MCT oil and monostearin gelator successfully stabilized metastable curcuminoids [16]. In comparison, the electrospinning encapsulation technique for phytochemicals has multiple advantages like straightforward one-step processing, high loading efficiency, dry product for convenient storage and applications, etc. However, few studies have been carried out on TC with this technique. Cellulose acetate phthalate and polyethylene glycol were once utilized for electrospinning encapsulation of TC, with successful in vitro release [17]. Curcumin-encapsulated electrospun materials have apparently been more researched. Recently, a fast-dissolving electrospun polysaccharide fibrous film loaded with curcumin was fabricated for the fast delivery of curcumin in the mouth [18]. A polylactic acid-based electrospun composite material loaded with curcumin was reported to achieve antibacterial properties against *E. coli* and *S. aureus* [19]. An electrospun polyvinylpyrrolidone and soy protein composite encapsulating curcumin nanoparticles was reported to possess hemocompatibility and antipathogenic activities, and wound-healing potentials [20]. The general trend of curcuminoid-loaded electrospun products was to achieve local effects like antibacterial [21,22,23] and wound healing [24,25,26]. However, they did not carry the bioactive compounds into the digestive tract to address the issues of low bioaccessibility and low absorption into the blood stream, which is important for exerting the health beneficial functions of the bioactive compounds [27,28].

In this research, an electrospun lipid–polymer composite (ELPC) was developed with high potential in addressing the bioaccessibility issue of TC. Completely GRAS level components were utilized that could safely assist the emulsification of TC in the digestive system. Gelatin was chosen as the backbone polymer of the ELPC due to its high availability and ease of electrospinning. MCT oil and lecithin were adopted in order to grant the ELPC an ability of self-assembling into emulsions to enhance TC delivery. In vitro assays including USP-4 dissolution, lipolysis, caco-2 cell colon membrane transport, caco-2 cytotoxicity and cellular uptake assays, which generally followed the order of gastrointestinal tract digestion, were adopted to evaluate the accessibility and related information on the TC-loaded ELPC.

## 2. Materials and Methods

### 2.1. Materials

Gelatin (from bovine skin, type B, ~225 Bloom), pancreatin (from porcine, P7545) and glacial acetic acid were purchased from Sigma Aldrich (St. Louis, MO, USA). Tetrahydrocurcumin was from Sichuan Weikeqi Biological Technology Co., Ltd. (batch# wkq22060710, purity > 98%, Chengdu, China). Medium chain triglyceride was from Stepan^®^ Company (Northfield, IL, USA) (Neobee 1053, triglycerides of 55% caprylic (C:8) and 44% capric (C:10) fatty acids). Lecithin was purchased from American Lecithin Company (Oxford, CT, USA) (LIPOID^®^ P 75, 70% phosphatidylcholine). 

### 2.2. Fabrication of the ELPC

Gelatin 16% (*w*/*v*), MCT 5.5% (*w*/*v*), PC-75 lecithin 5.5% (*w*/*v*) and TC 1.5% (*w*/*v*) were weighed before preparing the polymer blend in glacial acetic acid. Gelatin needs to be dissolved in glacial acetic acid at the very first step since it requires heating to dissolve (60–100 °C). MCT, lecithin and TC were then added to the solution and vortexed thoroughly to create a polymer blend for electrospinning. Before fabrication, the conductivity of the polymer blend was found to be 45.5 μS/cm at the temperature of 26.7 °C, while the surface tension was detected to be around 18.76 mN/m. 

**Figure 2:**
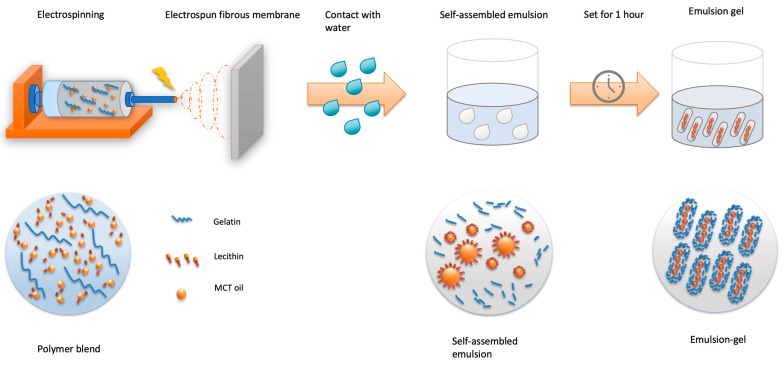
Schematics diagram for generation of the ELPC self-assembled emulsion and emulsion gel.

The polymer blend was then brought to electrospin. The electrospinning system was built manually and included a syringe pump (NEW ERA PUMP SYSTEMS NE-8000, Farmingdale, NY, USA), a high-voltage power supply (Glassman High Voltage Regulated HV DC Power Supply, Analogue—30 kV 5 mA, Somerset, NJ, USA), electric wires and a grounded aluminum foil-wrapped collector board. The feeding rate of the solution was set at 1 mL/h at the syringe pump. The distance between the grounded foil and the tip was 20 cm, and the electric field was set at 18 KV. After two hours of electrospinning, the ELPC film products were collected and placed in the fume hood with a strong airflow for 12 h to evaporate solvent residues and then kept in desiccators. The ELPC products were later placed in contact with water to generate self-assembled emulsions and set for one hour to become emulsion gels for further experiments. The whole process is illustrated in Figure 2. 

### 2.3. Morphology and Physical Characterizations

The morphology of the electrospun ELPC fiber films (n > 200) was captured by CLSM (Zeiss LSM 710, Jena, Germany), and average fiber diameters were measured by FIJI (Fiji Is Just ImageJ, version 1.0) software [29]. The self-assembled emulsion system was complicated (a mixture of the ELPC residue particles and oil droplets) and thus unable to be characterized by the widely used dynamic light scattering method; biases and errors had been detected. In order to estimate the emulsion droplet size distribution, CLSM was utilized again. The droplet sizes of the self-assembled nano-emulsions were also analyzed by FIJI. 

Due to the nature of gelatin, this self-assembled emulsion was able to set and become a gel when the ELPC concentration was high enough (tested > 4% by weight) and kept at room temperature or below. This gel could be defined as an emulsion gel since it fell into both categories. It has a dispersed phase (MCT + lecithin + TC) existing in a continuous phase matrix (gelatin + water). In order to obtain the detail structure of the hydrophilic and the hydrophobic components, for this part, the polymer blend was fluorescently dyed with 1% *w*/*v* coumarin-6, while the deionized water for generating the self-assembled emulsion was dyed with a hydrophilic fluorescent dye, rhodamine-6G at 1% *w*/*v*. By this means, the distribution between varied components within the special emulsion gel could be observed by CLSM.

### 2.4. Functional Characterizations

#### 2.4.1. Dissolution Profile for the ELPC

A USP type 4 apparatus (CE 7 smart manual closed loop) was adopted as the dissolution testing device for mimicking and analyzing dissolution of the ELPC in the stomach and intestine. This dissolution tester contains sample chamber cells filled with ruby and glass beads to mimic the movements of food inside the stomach. The dissolution medium was designed as 900 mL 0.1 N HCl (pH = 1, mimicking stomach juice acidity) for the first two hours and brought up to pH 6.8 by adding 100 mL basic buffered supplement (6.805 g/L KH_2_PO_4_ + 0.896 g/L NaOH) in the later hours to mimic the emptying of stomach and filling of colon [30]. The medium contained 1% tween 80, which was in accord with the FDA guideline for dissolution medium design. For the dissolution samples, the tested group was 1 mL water emulsified with 333.3 mg ELPC (containing 5 mg TC). The control group was 1 mL unformulated MCT oil suspension containing the same amount of 5 mg TC and 18.3 mg lecithin PC75 as the ELPC. The dissolution assay was carried out for 6 h totally at 37 °C. At the time intervals including 15 min, 0.5 h, 1 h, 2 h, 3 h, 4 h and 6 h, 0.5 mL of the dissolution medium was pipetted and mixed with 0.5 mL ethanol to break the emulsion-encapsulated TC for thorough detection. The 1 mL sample was then send to HPLC to assay the TC concentration and plot the dissolution profile curve for the ELPC.

#### 2.4.2. HPLC Detection of TC

Quantification of the TC amount in the dissolution samples was carried out with an Agilent 1100 series HPLC with a DAD detector and auto-sampler installed (Agilent Technologies, Santa Clara, CA, USA). A reverse-phase C18 column (ZORBAX SB-C18, 4.6 × 250 mm, 5-micron particle size) was utilized for the separation and identification of TC. The mobile phase was designed to run isocratically at a flow rate of 1 mL/minute with the composition of 60% acetonitrile and 40% of deionized-water with 0.1% of phosphoric acid added for reduction of tailing and better elution. The injection volume was 10 μL and the samples were filtered by 13 mm 0.22 μm Nylon Syringe filters (ArkBio Group Inc., Singapore) before HPLC sampling. The examination UV wavelength was set at 280 nm. Also, 425 nm was detected in the Caco-2 monolayer transport section as an internal standard. 

#### 2.4.3. In Vitro Lipid Digestion Assay by Lipolysis Experiment

The lipolysis experiment was designed to mimic the fed state and fasted state of humans to evaluate lipid digestion situations and bioaccessibility. It was achieved by preparing two types of buffering systems, as shown in Table 1.

The detail procedures of the lipolysis experiment are described as follows. Firstly, 1 g of pancreatin was dissolved in 5 mL of lipolysis buffer, which was heated to 37 °C and kept stirring for 15 minutes for thorough suspending. Then, the pancreatin suspension was centrifuged at 2000 rpm for another 15 min to separate the undissolved sediments. The supernatant, which was the pancreatin solution, was transferred to clean containers and chilled in ice for later use. Then, a 1.316 g ELPC sample containing 250 mg of MCT oil fraction, 250 mg lecithin and 0.066 g TC was added to 9 mL of lipolysis buffer and kept stirring at 37 °C for 10 min. The pH of the mixture was adjusted to 7.5 with 0.25 M sodium hydroxide solution drop by drop carefully and the amount of NaOH added was recorded all the time. When the pH reached 7.5, 1 mL of the pancreatin buffered solution prepared before was added quickly and then the titration experiment was started using 0.25 M sodium hydroxide solution drop by drop carefully. Under the effect of the pancreatin, triglycerides were broken down into free fatty acids and the TC was released, forming micelles with the surfactants in the matrix to become accessible. During this process, the free fatty acids kept dropping the pH of the system. As a result, titration of the free fatty acids could assay the amount of oil that was digested. The pH during the titration experiment was maintained at 7.5 ± 0.02 for a total of two hours until the end of the experiment. After that, the titrated mixture was chilled in ice and prepared for centrifugation. The parameter for ultracentrifugation was 50,000 rpm for 40 min at 4 °C. As the ultracentrifugation finished, the middle layer in the tubes was transferred out and filtered with a 0.2 μm filter. Then, 200 μL of the filtered sample was mixed well with 400 μL methanol and then brought to HPLC for detection of the final TC concentration.

In this section, both the lipolysis extent and bioaccessibility of TC from the ELPC were analyzed according to the previous reports [16]. For the extent of lipolysis, it was calculated as total NaOH used in titration/maximum amount of NaOH the lipid reacted with (2 × molarity of lipids) × 100%, assuming the reaction molarity ratio of NaOH and lipid molecule is two to one according to the enzyme properties. The bioaccessibility was calculated by the amount of free released TC in the centrifuged supernatant/the total amount of TC loaded in the sample × 100%. Because the molecular weight of the MCT was not readily determined, it could be estimated by saponification value, which is 334 for MCT. Then the molecular weight of the MCT could be calculated as 3 × 1000 × Mw KOH/334, which is about 504.

#### 2.4.4. Caco-2 Cell Culturing

To study the interaction between the formulated TC and human GI tract, multiple cell studies utilizing colon cancer cell line Caco-2 (ATCC HTB-37) were carried out, including Caco-2 monolayer membrane transport for bioaccessibility evaluation, Caco-2 cytotoxicity assay and cellular uptake studies. Caco-2 cells were cultured in DMEM with 10% fetal bovine serum, 100 IU/mL of penicillin and 100 mg/mL of streptomycin (Gibco™, Thermo Fisher Scientific, Waltham, MA, USA) at 37 °C with 5% CO_2_. Culturing medium was changed twice a week.

#### 2.4.5. Caco-2 Monolayer Membrane Transport Assay

In order to develop the monolayer membrane, the Caco-2 cells were seeded into 12-well plates utilizing specially designed inserts with 0.45 μM filters. Each insert was filled with 0.5 mL of cell suspension with a density of 0.6 × 10^6^ cells/mL. Each well was filled with 1.5 mL culture media mentioned in the previous section, and the media were changed every other day for both the inserts and the wells. The Caco-2 cells were cultured for about 21 days to reach an appropriate thickness for the transport assay of the TC-loaded ELPC samples. When the Caco-2 monolayer membrane was fully grown for the transport experiment, the media above and below the membrane were carefully removed from the edge of the container. Then, the membrane was cleaned using Hank’s balanced salt solution (HBSS) plus 25 mM HEPES (4-(2-hydroxyethyl)-1-piperazineethanesulfonic acid, a zwitterionic buffer) three times. After washing, the top insert was filled with 0.5 mL HBSS + HEPES while the bottom was filled with 1.5 mL, and they were maintained at 37 °C for half an hour before addition of the lipolyzed formulation. Then, transepithelial electrical resistance (TEER) was utilized to evaluate the quality of the monolayer, related to its thickness and integrity. The TEER value was measured by an EVOM2 Epithelial Voltohmmeter (World Precision Instruments). The appropriate thickness was defined as the TEER reading result, about 300 Ω. As long as the resistance reading was appropriate, the sample could be added to the compartment and the transport experiment started [31].

After electrical resistance evaluation, 5 μL of the sample was added into the top insert. The sample used here was the lipolyzed product of TC formulation (self-assembled TC emulsion from the ELPC). In detail, it was the product right after the lipolysis assay but before the titration step. In this part, only the fed state lipolyzed product was applied because sodium taurodeoxycholate is toxic to the Caco-2 cells. Samples were diluted about 100 times before application. For the control, TC solution in DMSO was prepared and adjusted to the concentration of the diluted lipolyzed TC formulation. The transport experiment was designed to run for 20, 40, 60, 80, 100 and 120 min. At each time interval, medium from the top insert and below was transferred to 2 mL centrifuge tubes. It was extracted with 1 mL ethyl acetate for 5 min three times and finally re-dissolved with 0.1 mL methanol to be detected by HPLC.

Intestinal membrane permeation and absorption were evaluated using the apparent permeation rate (P_app_). This is calculated as:

$$P_{app}=(\frac{dQ}{dt})(\frac{1}{AC_{0}})$$

where Q is the quantity of permeated substance, t is the interval, A is the permeation contact area = 1.1 cm^2^ and C_0_ is the concentration of substance at the beginning [32].

#### 2.4.6. Cytotoxicity Evaluation

To estimate the safety dosage of TC and the TC-loaded self-assembled ELPC nano-emulsion in the intestine, cytotoxicity of Caco-2 cells against these substances was examined. As previously mentioned, an MTT cell viability assay was conducted against the Caco-2 cells in a 96-well plate. Then, 1 × 10^4^ cells in the DMEM medium were seeded in each well and incubated at 37 °C for 24 h. On the next day, the medium was removed and the wells were washed with PBS 1X three times then filled with empty medium again. Then, 100 μL 0.5% DMSO solution of TC with concentration gradients was added to the wells. The control background was pure 0.5% DMSO 100 μL. The whole plate was then incubated in the environment same as above for another 24 h and then the medium was discarded and the plate was washed with PBS three times. After this, 100 μL MTT solution (0.5 mg/mL in RPMI 1640 media) was added to each well of the experiment and the plate was incubated at 37 °C for 2 h, and then the solution was all discarded and the plate was washed three times. After this, 100 μL DMSO was added to each well to dissolve the formazan produced by the living cells. Finally, the plate was fed to the microplate reader (Bio-Tek) to shake for 20 min, and the absorbance was read at 570 nm (490 nm as reference). Experiments were carried out in triplicate. Cell viability was defined as the percentage of the sample’s absorbance result divided by that of the control (background deducted).

#### 2.4.7. Cellular Uptake of Formulated TC Visualized by CLSM

In order to substantiate that the TC-loaded formulation was capable of entering the cells in the intestine as a final step in phytochemical absorption, a cellular uptake study was performed against the Caco-2 cell line. The Caco-2 cells were seeded on 20 mm round cover glasses (CELLTREAT^®^ SCIENTIFIC PRODUCTS, Pepperell, MA, USA) with a quantity of 1 × 10^5^ cells/mL and placed into 12-well plates. To visualize and semi-quantify the uptake situation for the formulated TC, the ELPC formulation was incorporated with 0.01% coumarin-6 dye as a fluorescence indicator. Then, 0.1 g of this fluorescent ELPC was put into 0.9 mL of cell culture medium to self-emulsify. This emulsion was further diluted with culture medium 500 times to ensure the coumarin-6 and TC amounts were safe for the cells. Then, the diluted emulsion medium was added to the wells for 0.5, 1, 2, 4 and 6 h to gradually observe the cellular uptake process. Another fluorescent dye, DAPI (10 μg/mL), was utilized for staining the nuclei area of the cells. At each time interval, medium in the wells was removed and DAPI solution was added to the wells to stain for 30 min. After that, the dye was washed away with PBS buffer three times to avoid over staining. The seeded glasses treated with emulsion and dyes were then brought to CLSM for examination. Addition of emulsion medium to the cells followed a reverse chronological order to allow all the time intervals to finish at the same moment, which facilitated CLSM examination. To capture images that could semi-quantify the accumulated active compounds within the cells, CLSM was dedicatedly tuned and configured with the same set of parameters throughout the whole imaging process. A 405 nm laser was used for the visualization of DAPI, while a 458 nm beam was used for exciting coumarin-6. The laser intensity of 405 nm was set to 0.03, and 0.025 for 458 nm for the best visual effects. The pinhole was set to 64.2 units to scan a 17.9 μm section. For the DAPI channel, the gain was set to 575 units, while for the Nile red channel the gain was 776.

#### 2.4.8. Statistical Analysis

The means, standard deviations and variance analysis of presented data were conducted with OriginPro Learning Edition. Particle diameters, CLSM image processing and intensity evaluation were performed using Fiji software (version 1.0) [29]. Mean values were compared and statistical significance was accepted at a 0.05 confidence level.

## 3. Results

### 3.1. Physical Characterizations

#### 3.1.1. Morphology of the ELPC Product

**Figure 3:**
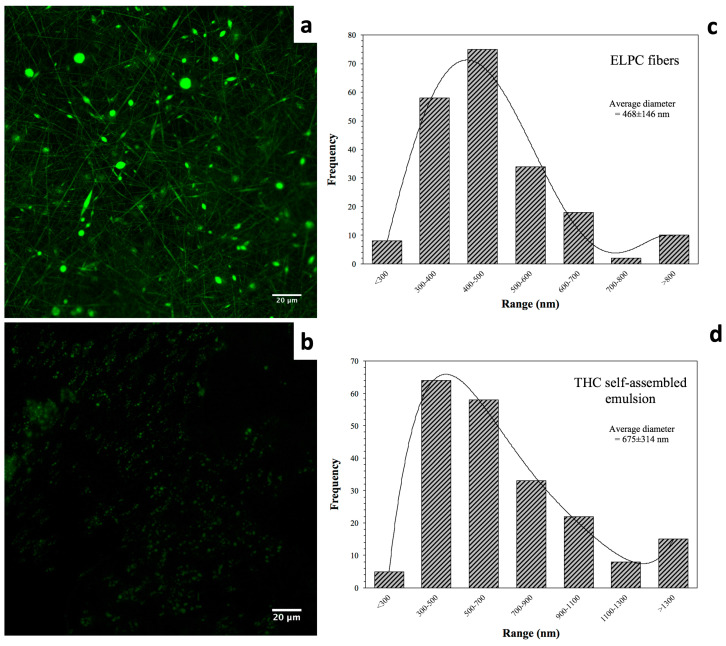
(**a**) CLSM microscopic view of the TC-loaded ELPC fibrous film dyed with coumarin-6. (**b**) CLSM image of the self-assembled emulsion generated by the TC-loaded ELPC. (**c**) Diameter distribution of the TC-loaded ELPC electrospun fibers. (**d**) Diameter distribution of the TC emulsion droplets generated from ELPC.

Morphology of the electrospun composite is displayed in Figure 3a. A green color was applied to indicate the fluorescence of the electrospun ELPC fibers. In the image, both fine fibers and small particles were observed. This implies that the fabrication process was somewhere between electrospinning (fiber forming) and electrospraying (particle forming). It could be observed that the fiber diameter distribution was relatively even. As shown in the chart in Figure 3c, the fibers had a relatively focused diameter distribution ranging from 300 to 700 nanometers. Also, 67% of the fibers were 300–500 nm in diameter and the average diameter of all fibers was 468 ± 146 nm (200 pieces of fibers measured).

#### 3.1.2. The ELPC-Generated Self-Assembled Emulsion Examined by CLSM

CLSM visualizing the self-assembled ELPC emulsion is demonstrated in Figure 3b. FIJI software analysis reported that the average diameter of the droplets was around 675 nm (200 droplets were measured, distribution data in Figure 3d). 

#### 3.1.3. Structure of the ELPC-Generated Self-Assembled Emulsion Gel Examined by CLSM

**Figure 4:**
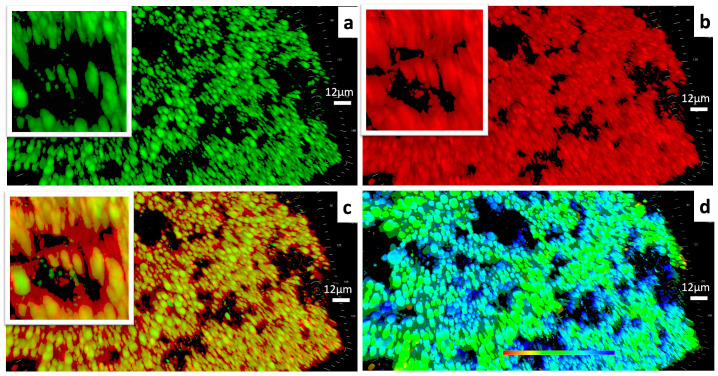
(**a**) CLSM images of the lipophilic components within the ELPC emulsion gel. Lipophilic contents dyed with coumarin-6 are labeled in green. (**b**) CLSM images of the hydrophilic components within the ELPC emulsion gel. Hydrophilic contents dyed with Rhodamine-6G are labeled in red. (**c**) Combined image displaying the distribution of lipophilic/hydrophilic components within the ELPC emulsion gel. (**d**) Phase image of the emulsion gel (The shallow to the deep phase was represented by the red to the blue in color).

As Figure 4 depicts, the gel framework had a massive clustered structure built with capsule-like units. It could be observed that the lipophilic contents were encapsulated within each hydrophilic “capsule” independently. According to the 3-D scale bar and the phase image, the capsule like units were about 3–6 microns in width and up to about 22.41 microns in height. 

#### 3.1.4. FTIR Examination

**Figure 5:**
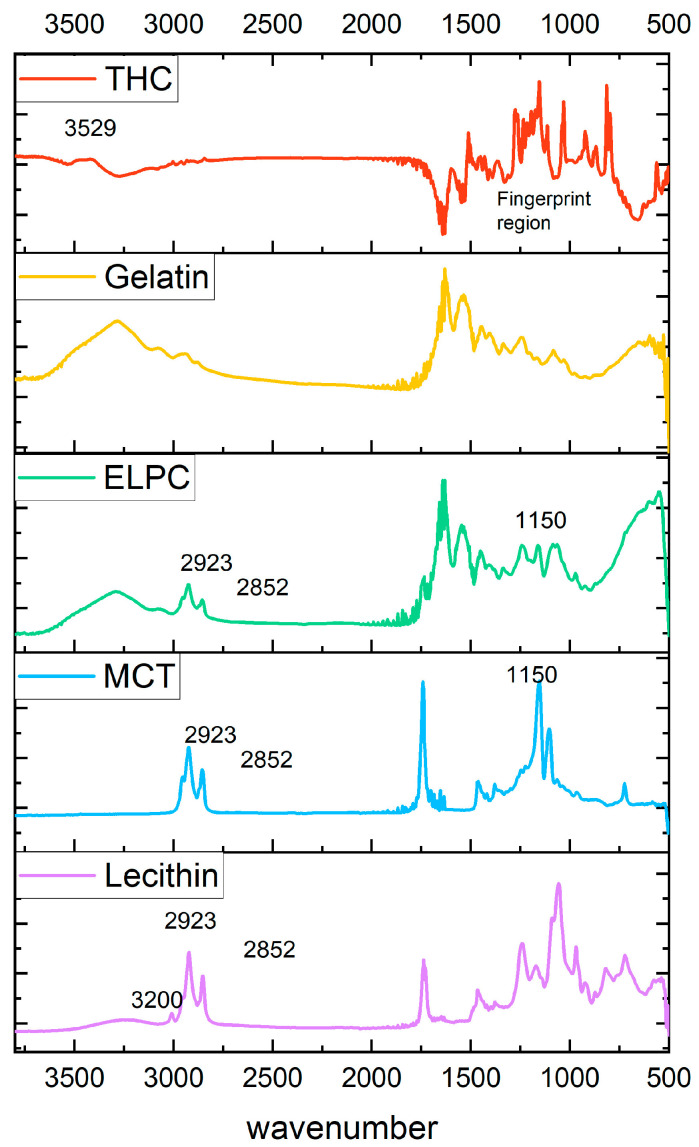
FTIR spectrums of varied components in the ELPC.

Information about functional groups of varied components in the ELPC was examined by FTIR, with data shown in Figure 5. TC crystal has a fingerprint pattern region in the wavenumber range 500–1600 cm^−1^. Nevertheless, these fingerprint signals disappeared in the ELPC loaded with TC, which was in accord with previous reports [33,34]. IR peaks at 2923 and 2852 cm^−1^ for the alkyl C-H stretch on the spectrum represented the hydrocarbons in the alkyl chain of lecithin and MCT oil [35]. These C-H stretch signals were also found in the ELPC. In addition, a strong C-O stretch at a wavenumber of 1150 from the ester bond of the large amount of MCT oil was detected in the ELPC with MCT, which was not observed in the FTIR spectrum of pure gelatin. These clues are potentially correlated with the fact that the lipids in the ELPC exist in some degree of crystalline form. A strong and broad hydrogen-bonded O-H stretch at the wavenumber 3200–3600 cm^−1^ was displayed in the IR spectrum for lecithin PC75. However, this signal was significantly reduced when lecithin was incorporated within the ELPC, implying that intermolecular hydrogen bonding of lecithin was disrupted and it was not a major interaction mechanism between the components within the ELPC. Hydrophobic interactions and Van der Waals forces were dominant interactions inside the massive ELPC matrix.

### 3.2. Functional Characterizations for the ELPC

#### 3.2.1. Dissolution of the ELPC

**Figure 6:**
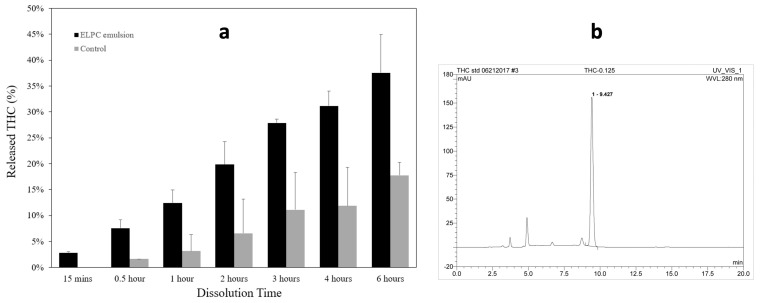
(**a**) Dissolution profile of the TC−loaded ELPC compared with the TC oil suspension. (**b**) HPLC chromatogram of TC.

Dissolution profiles of the TC-loaded self-assembled emulsion versus TC oil suspension as the control are displayed in Figure 6a. Dissolute TC was detected by HPLC with the chromatogram displayed in Figure 6b, and the retention time of TC was 9.4 min. Both the emulsion and the suspension contained 5 mg of TC, and the released percentage of TC from emulsion or suspension were plotted together. For the first two hours (pH = 1), the TC dissolution amount from the ELPC emulsion was 3 to 4.6 times higher than the oil suspension; for the later four hours (pH = 6.8), the ELPC still outperformed the control group by 2.1 to 2.6-fold. 

#### 3.2.2. In Vitro Lipid Digestion Assay by Lipolysis

**Figure 7:**
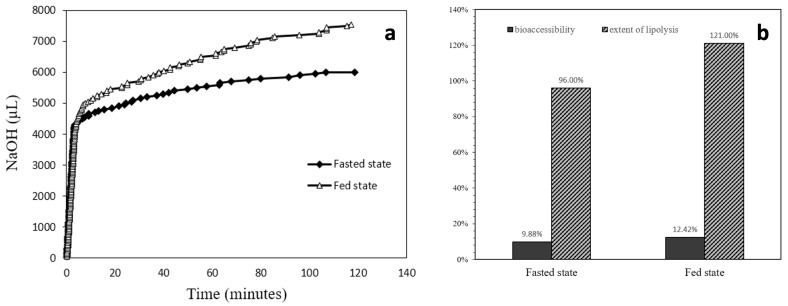
(**a**) Lipolysis profiles comparison between fasted state and fed state situations. (**b**) Bioaccessibility and extent of lipolysis of the ELPC in varied situations.

From the lipid digestion profile displayed in Figure 7a, it was apparent that the speed of digestion in the first several minutes was rapid. The log phase for the fed state digestion lasted about 6.75 min, while for the fasted state the rapid digestion lasted about 3.35 min. During the log phase of digestion, the fed state situation digested 65% of the total lipolyzed lipids; for the fasted state, 72% total lipolyzed lipids was consumed. The chart in Figure 7b displays both the calculated bioaccessibility and the extent of lipolysis for fasted and fed state situations. The bioaccessibility was 9.88% for the fasted state and was increased to 12.42% for the fed state. The lipolysis extent was 96% for the fasted state and 121% for the fed state.

#### 3.2.3. Caco-2 Monolayer Membrane Transport Assay

**Figure 8:**
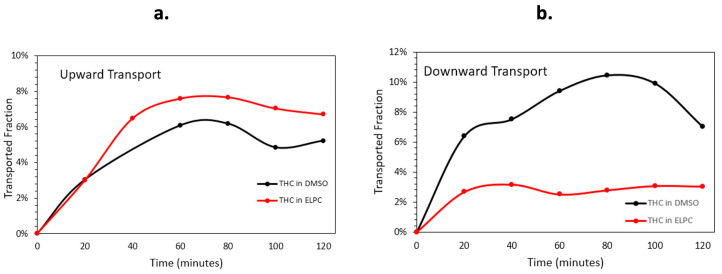
(**a**) Upward and (**b**) downward transportation for TC in DMSO solution and TC in the lipolyzed ELPC.

The Caco-2 monolayer membrane transportation data are shown in Figure 8. Quantity and speed of the Caco-2 membrane transport varied apparently in different transport directions. For the upwards transport (basolateral to apical), the lipolyzed ELPC had a slight advantage of permeation performance over the TC DMSO solution. Nevertheless, when the situation turned into downward transport (apical to basolateral), the ELPC no longer outperformed the DMSO solution. The overall downward transport amount of the lipolyzed ELPC was about one third of the TC in DMSO solution, and the amount was much lower than for upward transport. In addition, it could be observed that the general trend of transport was firstly increased for 80 min then decreased until the end of the experiment. 

#### 3.2.4. MTT Assay for TC and the TC-Loaded ELPC Emulsion

**Figure 9:**
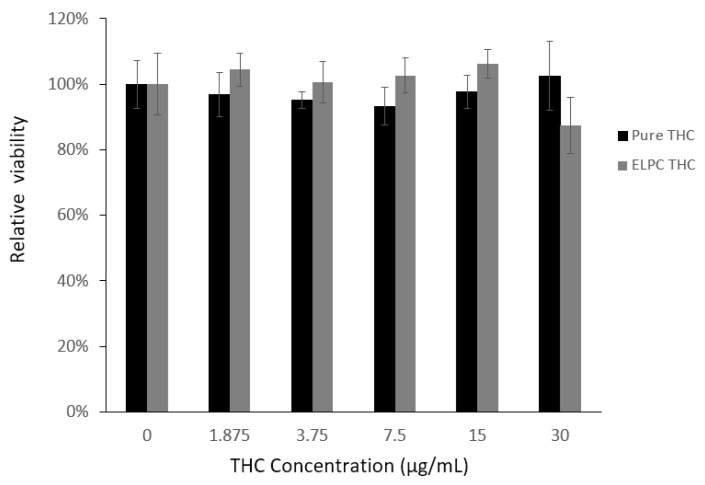
MTT assay for the cell viability against pure TC and the ELPC emulsion TC.

As demonstrated in Figure 9, the overall cytotoxicity of TC against Caco-2 cells was low. All the designed TC concentrations from 0 to 30 μg/mL were considered non-toxic to Caco-2 cells (viability > 90%). For the TC loaded in the ELPC self-assembled emulsions, this was also non-toxic and even slightly promoted the growth of the cells, except the 30 μg/mL concentration where the cytotoxicity was right around the safety line (88%). As a result, TC in the ELPC emulsions up to 15 μg /mL was confirmed safe for the following cellular uptake study.

#### 3.2.5. Cellular Uptake for TC and the TC-Loaded ELPC Emulsion

**Figure 10:**
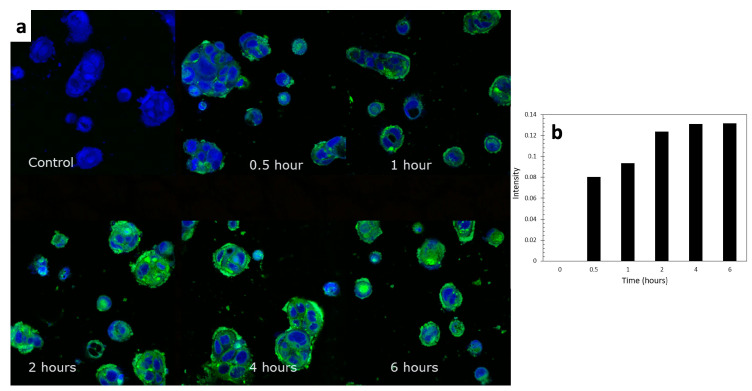
(**a**) CLSM images of the cellular uptake process of the TC emulsion self-assembled from the ELPC over 6 h. (**b**) Semi-quantification of the cellular uptake.

From the CLSM images in Figure 10a, Caco-2 cellular uptake of TC was clearly observed as the accumulation of the green channel color throughout the designed range of time. It could also be observed that the ELPC emulsion accumulated throughout the cytoplasm but especially a higher amount was seen in the membranes of the Caco-2 cells. The uptake process was relatively rapid. The increase in relative fluorescence intensity (background deducted) was plotted against time in the chart demonstrated in Figure 10b, which was a semi-quantification of the cellular uptake rate. It could be concluded from the chart that it just took 30 min for the Caco-2 cells to achieve about 61% of the equilibrium level of TC uptake. From two hours on, the uptake process approached the equilibrium and reached the peak at around 4 h.

## 4. Discussion

The ELPC was designed to enhance TC dissolution and absorption in the digestive tract. These assumptive effects were preliminarily supported by the results from a series of in vitro characterizations in this research. However, if the imperfections in the ELPC could be improved, the dissolution and absorption results could be more satisfying. Microscopic observation of the ELPC displayed both fibers and beads instead of stable bead-free fibers. In addition, the self-assembled emulsion had a relatively large average droplet size around 675 nm, which is apparently larger than the droplets of routine nano-emulsions [36,37,38]. These observations implied that further tuning of the ELPC formulation was necessary, which could possibly promote the delivery of TC. 

The emulsion gel generated by the ELPC was found to be a large and clustered gel matrix constructed by numerous micron-scale “capsule units”. The lipid phase appeared to be spheres or rods with round ends, which were encapsulated inside each “capsule unit” instead of existing in the hollow spaces of the gelatin matrix. The mechanism behind their round appearance was that they had the lowest surface-to-volume ratio, lowest surface energy and highest interfacial stability against the hydrophilic gelatin “capsule shell”. 

FTIR results displayed that the fingerprint spectrum of crystalline TC was concealed in the ELPC; however, the hydrocarbon stretch from the incorporated lipids was found. Detailed interactions and physical status of various components inside the ELPC remain to be researched. USP-4 dissolution results substantiated that the ELPC demonstrated a high performance in delivering TC through the GI tract, which was a 2.1 to 4.6-fold increased delivery over the same amount of TC-in-oil suspension. Because this was a closed system apparatus, a sink condition could not be achieved. In addition, the medium contained only 1% tween 80 (USP requirement), which limited the solubility of TC. Considering this limitation, if the ELPC was consumed orally, the dissolution of TC should be potentially better, with higher amounts of digestive fluid involved. 

Lipolysis results revealed that the lipid inside the ELPC was digested at a fast pace in less than 10 min. However, the bioaccessibility of TC in the ELPC was relatively low, which might be related to the insufficient self-emulsifying of the ELPC that formed aggregates with low bioaccessibility. The aggregation might also contribute to the relatively large droplet size of the self-assembled ELPC emulsion (>600 nm). Another explanation could be that the interaction between TC and gelatin was strong, which retarded the release. In the fed state situation, lipid would be digested more completely and higher amounts of TC could be released. This observation result agreed with a previous report [39], indicating that ELPC would perform better if consumed after meals. 

Caco-2 monolayer membrane transport results confirmed the intestinal delivery of TC from the ELPC. In the upward transport direction, the ELPC outperformed the DMSO solution of TC. Nevertheless, this advantage was not continued in the downward direction. Usually, the absorptive transport (downward) amount was higher and faster than the secretory transport (upward) [40,41]. However, the ELPC transported more TC in the upward direction than downwards. It could be inferred that a certain amount of TC formulation was absorbed and entrapped within the intestine cells during the absorptive downward transport, which was supported by the cellular uptake images. During downward transport, the sample would firstly contact the microvilli of the monolayer. The filtering ability of the microvilli would further lower the amount of the downward transported TC formulation. 

Studies of cytotoxicity found that the raw TC was generally non-toxic to the Caco-2 cells (viability > 90%). Interestingly, when the TC-loaded ELPC emulsion was applied at concentrations no more than 15 μg/mL, the cell viability achieved over 100%. This means that the formulated TC was not only non-toxic but also promotive to cell growth. The reason could be that the lecithin and gelatin in the ELPC were nutritious for the cells. Cellular uptake images demonstrated rapid delivery of TC into the Caco-2 cells by the ELPC self-assembled emulsions. This fast absorption effect could help to explain the decline in TC transport at the later stage of the Caco-2 monolayer assay, and might also be the reason behind the compromised downward transport performance of the ELPC. The CLSM images also displayed that the formulation tended to accumulate more at the edges of the Caco-2 cells, since MCT oil and lecithin in the ELPC had high affinity to the cell membranes that were rich in phospholipids. As time went by, more and larger fluorescent aggregates were found outside the cells, which could be the process of cellular excretion of the formulation.

## 5. Conclusions

In this research, an electrospun lipid–polymer composite delivery system (ELPC) loaded with TC was developed with whole GRAS components. CLSM fluorescence microscopy found that the ELPC was composed mainly by electrospun fibers with an average diameter of 468 ± 146 nm. The ELPC could generate self-assembled emulsion with droplet sizes around 675 ± 314 nm. The ELPC emulsion could form a gel constructed by numerous capsule-like units clustering together under CLSM observation. The lipid phase of ELPC was entrapped inside each “capsule”, with rod-like shapes to reduce its surface energy. The FTIR fingerprint spectrum of TC was not observed in the ELPC, while the hydrocarbon stretch from the incorporated lipids was found, implying that the loaded TC could be amorphous while the lipid phase might have a certain degree of crystallinity in the ELPC. Dissolution profiles substantiated that the ELPC had a 2.1 to 4.6-fold increased TC delivery over oil suspension. The lipolysis assay proved that the lipid inside ELPC was digested rapidly. In the upward transport of the Caco-2 monolayer membrane assay, ELPC appeared with high transport ability that outperformed the TC DMSO solution. Nevertheless, this advantage was not seen downwards, which might due to the microvilli filtration effect and cellular uptake. Cellular uptake results demonstrated a rapid TC delivery to the cells by ELPC that just took 30 min to uptake 61% of the maximum amount. In a word, the ELPC has high potential in assisting the delivery of hydrophobic bioactive compounds. Future research should focus on tuning the ELPC polymer blend formulations to achieve better encapsulation efficiency and lower droplet size in the self-assembled emulsion, which might further increase the bioaccessibility of bioactive compounds.

## Figures and Tables

**Table 1:** Recipes for fed and fasted state buffer systems, measured in gram/liter.

Ingredients	Fed	Fasted
Tris Maleate	11.86	11.86
NaCl	8.7664	8.7664
CaCl_2_·2H_2_O	0.7351	0.7351
NaTDC (sodium taurodeoxycholate, bile salts)	2.6084	10.4336
Phosphatidylcholine	0.9501	3.8004

## Data Availability

The original contributions presented in the study are included in the article, further inquiries can be directed to the corresponding authors.
